# Surgical results and quality of life of patients submitted to restorative proctocolectomy and ileal pouch-anal anastomosis

**DOI:** 10.1590/0100-6991e-20202791

**Published:** 2021-03-17

**Authors:** ADRIANA CHEREM-ALVES, ANTÔNIO LACERDA-FILHO, PRISCILA FERNANDES ALVES, MAGDA PROFETA-DA-LUZ, JULIANO ALVES FIGUEIREDO, RODRIGO GOMES DA-SILVA

**Affiliations:** 1 - Hospital das Clínicas- UFMG, Instituto Alfa de Gastroenterologia - Belo Horizonte - MG - Brasil; 2 - Universidade Federal de Minas Gerais, Departamento de Cirurgia - Belo Horizonte - MG - Brasil

**Keywords:** Restorative Proctocolectomy, Ulcerative Colitis, Polyposis, Quality of Life, Morbidity, Mortality, Proctocolectomia, Retocolite Ulcerativa, Polipose Adenomatosa Familiar, Morbidade, Qualidade de Vida

## Abstract

**Purpose:**

restorative proctocolectomy with ileal pouch-anal anastomosis (IPAA) is the surgical procedure of choice in some cases of familial adenomatous polyposis (FAP) and ulcerative colitis (UC). IPAA allows complete removal of the diseased colon and rectum, however, it is associated with substantial morbidity and potential consequences to patients’ quality of life (QoL).

**Aims:**

to evaluate the surgical results, functional outcomes and QoL after IPAA; and to examine the impact of surgical complications upon QoL*.*

**Methods:**

we reviewed the records of 55 patients after IPAA, with emphasis on surgical outcomes. Forty patients answered the questionnaires. The Cleveland Global Quality of Life (CGQL), Inflammatory Bowel Disease Questionnaire (IBDQ), and Short Form 36 Health Survey Questionnaire (SF36).

**Results:**

the average age was 42.1±14.1 years. 63.6% of the patients were male, and 69.1% had FAP. Operative mortality was 1.8% and overall morbidity was 76.4%. Anastomotic leakage was the most frequent early complication (34.5%). Pouchitis (10.8%) and small bowel obstruction (9.1%) were the most common late complications. Patients with UC had the most severe complications (p=0.014). Pelvic complications did not have a negative effect on functional outcomes or QoL scores. Female patients had decreased pouch evacuation frequency, fewer nocturnal bowel movements, decreased bowel symptom impact on QoL (p=0.012), and better CGQL (p=0.04). Patients with better education had better QoL scores, and patients who had their pouches for more than five years scored lower.

**Conclusion:**

the high morbidity has no impact on function or QoL. Bowel function is generally acceptable. QoL is good and affected by sex, education and time interval since IPAA.

## RESUMO

**Objetivo:**

a Proctocolectomia com reservatório ileoanal (PCT-RIA) é método de escolha em alguns casos de Polipose Adenomatosa Familiar (PAF) e Retocolite Ulcerativa (RCU). Embora tenha potencial curativo, apresenta morbidade considerável e pode afetar a qualidade de vida (QV) dos pacientes.

**Objetivos:**

avaliar resultados cirúrgicos e impacto das complicações pélvicas na função intestinal e QV.

**Métodos:**

foram avaliados retrospectivamente 55 pacientes submetidos a PCT-RIA, de janeiro de 2003 até abril de 2017, com ênfase na técnica operatória e morbidade. Quarenta pacientes responderam aos questionários Cleveland Global Quality of Life (CGQL), Inflammatory Bowel Disease Questionnaire (IBDQ) e Short Form 36 Health Survey Questionnaire (SF36).

**Resultados:**

A média de idade foi 42,1±14,1 anos, sendo 63,6% do sexo masculino e 69,1% com diagnóstico de PAF. A mortalidade cirúrgica foi 1,8% e morbidade 76,4%. Fístula anastomótica foi a complicação precoce mais frequente (34,5%) e, as tardias foram bolsite (10,8%) e obstrução intestinal (9,1%). As complicações precoces mais graves foram mais frequentes em pacientes com RCU (p=0,014). Não houve impacto das complicações na função intestinal nem na QV. As mulheres apresentaram menor frequência evacuatória e noturna, menor interferência dos sintomas intestinais na QV (p=0,012) e CGQL mais elevado (p=0,04). Melhor QV foi referida pelos pacientes com maior escolaridade e, foi observada piora em pacientes com mais de cinco anos de confecção do RIA.

**Conclusões:**

não se evidenciou impacto das complicações na função intestinal nem na QV. A função intestinal é satisfatória e a QV é boa na maioria dos pacientes, sendo influenciada pelo sexo, escolaridade e tempo de confecção do RIA.

## INTRODUCTION

Proctocolectomy (PCT) with ileal pouch-anal anastomosis (IPAA) consists of the complete resection of the colon and rectum and preparation of a terminal ileum pouch, or reservoir, with anastomosis to the anus or up to 2 cm above the dentate line. It is currently the surgical technique of choice in cases of Familial Adenomatous Polyposis (FAP), in the classic or profuse form, and ulcerative colitis (UC) refractory to drug treatment or when malignancy occurs^1^. Its main advantage is the preservation of the natural evacuation route of the intestinal contents, avoiding definitive ileostomy. However, it is a procedure with extensive morbidity, both in terms of complications and functionality, with a considerable impact on Quality of Life (QoL)^1^.

Surgical complications can occur both perioperatively and late, and involve anastomotic fistulas, urinary fistulas, or fistulas to other pelvic organs, hemorrhages, strictures, adhesions, pouch failure, urinary and sexual dysfunctions, incisional hernias, and pouchitis^1^ ^-^ ^3^. The management and treatment of such complications usually impose a great challenge^4^, and their impact on the pouch function in the short and long term is not entirely known^5^ ^-^ ^8^.

In Brazil, IPAA has been carried out in tertiary centers, by specialized teams, for about 30 years. However, national publications on the subject are scarce^3^ ^,^ ^9^ ^-^ ^12^, especially with regards to morbidity and mortality, QoL, and pouch function. This study aims to evaluate the surgical results of IPAA, mainly in terms of complications, intestinal function, QoL, and respective relationships with the socio-demographic and clinical characteristics of patients with UC and FAP undergoing this procedure.

## METHODS

This is a cross-sectional, descriptive, and analytical study, in which we evaluated 67 patients with a diagnosis of UC or FAP who underwent PCT with J-shaped IPAA by the Coloproctology group at the Alfa Institute of Gastroenterology, Hospital das Clinicas, UFMG, from January 2003 to April 2017. We performed no sample calculation, considering all patients in that period who met the study’s inclusion and exclusion criteria.

We included patients over 18 years, who underwent PCT-IPAA, agreed to participate in the study, and signed an informed consent form.

We excluded patients with a presumed diagnosis of UC who later developed criteria for Crohn’s disease diagnosis, who were illiterate or unable to understand the terms of the study, those with incapacitating physical condition impairment, and patients with incomplete medical records.

For the assessment of pouchitis episodes, we excluded ileostomized patients (six individuals with definitive ileostomies due to IPAA failure and two with protective ileostomies) and one who died due to complications of ileostomy closure.

For the evaluation of IPAA function and QoL, we excluded ileostomized patients, the deceased, and those patients with less than a year of stoma closure.

**Figure 1:**
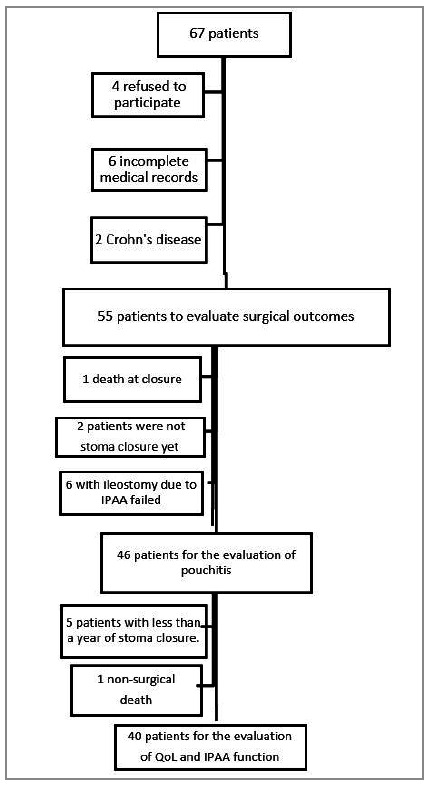
Flowchart of patient selection for outcome evaluation surgical, QoL and IPAA function.

After applying the inclusion and exclusion criteria, we included 55 patients for the analysis of surgical outcomes, 46 individuals for the evaluation of pouchitis, and 40 for QoL evaluation (Figure 1).

We collected data on socio-demographics, surgery indication, presence or absence of malignancy in the surgical specimen, surgical strategy (one, two or three steps), anastomosis type (stapled or manual), and surgical outcomes, including complications.

The operations were performed in one, two or three steps, depending on patients’ indication and clinical status. When performed in two stages, the first procedure consisted of the PCT-IPAA and a protective ileostomy, and in the second stage, the ileostomy was closed. In the three-stage surgery, the pelvic part of the surgery was performed after improvement of the patient’s clinical condition. Therefore, the first stage comprised subtotal colectomy with terminal ileostomy and mucous fistula of the sigmoid remnant; the second stage comprehended the protectomy with IPAA and protective ileostomy; and the closure of the ileostomy in the third stage.

We considered early complications those that occurred within 30 days of any of the surgical procedures, and the late ones, those that occurred after that period. We classified early complications according to the Clavien-Dindo surgical complications score^13^.

We considered an anastomotic fistula to be any defect in intestinal integrity at the anastomosis site leading to communication between the intra and extra-intestinal compartments. According to this classification, we also considered abscesses at the same site as the anastomosis to be fistulas^14^, analyzing them together.

Pelvic complications were the ones related to the proctectomy and the manufacturing of the IPAA, thus comprising the following: anastomotic stenosis, IPAA fistula to the vagina, pelvic abscess or anastomotic fistula, pelvic hemorrhage, stapling line bleeding, lesion of the distal ureter, and IPAA fistula to the bladder.

We classified IPAA failure as the need for permanent stoma, with or without IPAA excision^15^. For the other described complications, we considered the clinical suspicion as duly registered in the medical records and the results of complementary exams, when requested.

Surgical death was that which occurred at any time during the surgical procedure and within 30 days afterwards.

The time of IPAA was calculated between the time elapsed from the closing date of the ileostomy and the date of the interview to assess QoL.

We assessed QoL with 40 patients through face-to-face application of the Short Form Health Survey (SF36)^16^, the Inflammatory Bowel Disease Questionnaire (IBDQ)^17^, the Cleveland Global Quality of Life (CGQL)^18^, and questions specific to the IPAA function.

We also inquired patients specifically about the IPAA function, assessing urgency, incontinence, fecal leak, number of bowel movements in 24 hours and at night, food restriction, incomplete bowel movements, and the use of antidiarrheal medications. These questions, as well as the subjective question (“how do intestinal symptoms affect your quality of life”) were adapted from specific functional questionnaires^19^ ^-^ ^21^. As these questionnaires were not validated for Brazil, the calculation of the scores was not performed, only the description of results.

This study was approved by the Ethics in Research Committee of UFMG (COEP) - Project CAAE 50917215000005149.

### Statistical analysis

We computed the mean ± standard deviation (SD) for quantitative variables when their distribution was normal, and median (Q1;Q3) for non-normally-distributed variables. We described categorical variables with absolute frequency and percentage. For comparison between categorical variables, we performed the exact and asymptotic Chi-square Pearson tests.

For comparisons of the quantitative variables, if the variable was not normally distribution, we used the Mann Whitney and Kruskal Wallis tests. In the multiple comparison paired test after a significant Kruskal Wallis test, we used the Mann Whitney test with Bonferrone correction. The normal distribution test used was the Shapiro Wilk. In the analysis of correlations, we used the Spearman test.

The level of significance used was 0.05 and the software used was the Statistical Package for Social Sciences (SPSS).

## RESULTS

Of the 55 patients included, the majority were male (63.6%), with a mean age of 42.1 ± 14.1 years, and most (55.1%) had more than eight years of schooling.

Most patients had FAP (69.1%) and 26% of these had concomitant colon adenocarcinoma.

The main indication for IPAA in UC was clinical treatment failure (76.5%), followed by severe acute colitis (17.6%), and dysplasia (one patient - 5.9%). At the time of surgery indication, 94.1% of patients used corticosteroids, and 52.9%, anti TNF alpha drugs.

Regarding the surgical technique, most anastomoses were performed by double stapling (94.7%). The three-step procedure was performed in 58.8% of cases of UC, the remainder of patients undergoing two-step surgery. In FAP cases, all approaches were performed in two stages, except for one patient (1.8%) in whom it was not possible to perform an ileostomy due to technical difficulties related to the great thickness of the abdominal wall and a short mesentery.

Surgical morbidity was 76.4%, and mortality, 1.8%. Early complications occurred in 65.5% of patients, and late complications, in 41.8%.

The most frequent early complication was anastomotic fistula or pelvic abscess, in 19 patients (34.5%), with no statistically significant difference between patients with UC or FAP (Table 1).

**Table 1:** Results of the comparison of early surgical complications between patients with UC and with FAP undergoing PCT with IPAA (n = 55).

Variables	UC N=17	FAP N=38	p-Value
Pelvic hemorrhage	0 (0.0)	7 (18.4)	0.086^2^
Staple line bleeding	0 (0.0)	1 (2.6)	1.000^2^
Anastomotic fistula or pelvic abscess	3 (17.6)	16 (42.1)	0.078^1^
ITU	1 (5.9)	3 (7.9)	1.000^2^
Ureter injury	2 (11.8)	0 (0.0)	0.092^2^
DVT / TEP	1 (5.9)	1 (2.6)	1.000^2^
Early IPAA fistula for the vagina	1 (5.9)	1 (2.6)	1.000^2^
Paralytic ileus	1 (5.9)	3 (7.9)	1.000^2^
Wound infection	3 (17.6)	5 (13.2)	0.692^2^
Hemodynamic instability	1 (5.9)	2 (5.3)	1.000^2^
Urinary retention	2 (11.8)	2 (5.3)	0.580^2^
Others ¥	1 (5.9)	1 (2.6)	1.000^2^
Total patients with early complications	9 (52.9)	27 (71.1)	0.192^1^

^1^Asymptotic Pearson’s Chi-square test; ^2^Pearson’s exact chi-square test; ITU - Infection Tract Urinary; DVT - Deep Venous Thrombosis; TEP - Pulmonary thromboembolism; ¥ Others - Laparostomy, loboccipital ischemia and pancreatitis.

The total number of early complications per patient ranged from zero to four, with 15 patients (27.3%) presenting more than one complication. Most complications (64.4%) were managed conservatively (Clavien-Dindo I and II). When grouping the most serious complications (Clavien-Dindo IIIb, IV and V), we identified a higher occurrence in patients with UC than with FAP (Table 2).

**Table 2:** Result of the comparison of the total of early complications (n = 59) per patient and Clavien-Dindo classification between patients with FAP and with UC who underwent PCT with IPAA (n = 55).

Variables	UC	FAP	Total	p-Value¹
Total early complications per patient				
0	8 (47.1)	11 (28.9)	19	0.190
1	4 (23.5)	17 (44.7)	21	0.135
2	3 (17.6)	5 (13.2)	8	0.669
3	1 (5.9)	5 (13.2)	6	0.423
4	1 (5.9)	0 (0.0)	1	0.131
Total patients	17	38	55	
Clavien-Dindo classification				
Grade I	4 (23.5)	12 (28.6)	16	0.694
Grade II	5 (29.4)	17 (40.5)	22	0.431
Grade IIIa	1 (5.9)	8 (19.0)	9	0.208
Grade IIIb	3 (17.6)	5 (11.9)	8	0.570
Grade IVa	3 (17.6)	0 (0.0)	3	0.008
Grade V	1 (5.9)	0 (0.0)	1	0.131
Total early complications	17	42	59	
Grade IIIb+IVa+V	7 (41.2)	5 (11.9)	12	0.014

^1^Z proportion test.

The most frequent late complications were episodes of pouchitis and intestinal obstruction (10.8% and 9.1%, respectively), pouchitis being more frequent in patients with UC (Table 3). Two patients needed surgical intervention for intestinal obstruction, the others being handled clinically.

**Table 3:** Results of the comparison of late surgical complications between patients with UC and with FAP undergoing PCT with IPAA (n = 55).

Variables	UC	FAP	p-Value^1^
Late IPAA fistula for the vagina	0 (0.0)	3 (7.9)	0.544
IPAA fistula for the bladder	0 (0.0)	1 (2.6)	1.000
Anastomosis stenosis	0 (0.0)	2 (5.3)	0.565
Incisional hernia	2 (11.8)	3 (7.9)	1.000
Bowel obstruction	2 (11.8)	5 (13.2)	1.000
Pouchitis¥ (N=46)	6 (40.0)	1 (3.2)	0.003

^1^Pearson’s exact chi-square test; ¥ excluded ileostomized patients.

Pelvic complications occurred in 26 patients (47.3%) and corresponded to 44.0% of the total complications. There was no difference between diseases (UC and FAP) as for pelvic complications.

IPAA failure occurred in 10.9% of patients, all with FAP. The causes of failure were two cases of IPAA anastomotic fistula, two of IPAA fistulas to other organs (one to the vagina and one to the urinary bladder), and one mesentery desmoid tumor resection with compromised vasculature of the reservoir.

Two patients died, one who had FAP, of urothelial carcinoma of the bladder (non-surgical death), and the other with UC, who had fistula and fecal peritonitis after the closure of the protective ileostomy.

In the assessment of the QoL and IPAA function, 40 patients were included, 15 with UC and 25 with FAP. The mean age was 39.2 ± 13.1 years. Twenty-five patients were male (62.5%) and the IPAA time varied from one to 11 years, with a median of 3.75 years. The obstetric history showed that eight women (53.3%) were nulliparous and four (26.7%) had previous vaginal deliveries, the maximum of vaginal deliveries being two.

In the assessment of IPAA function (n = 40), the median of evacuations in 24 hours was six, one at night. Most patients (87.5%) did not have incontinence for solid stools, while 50% reported fecal leak. Evacuation urgency was reported by 32.5% of patients, with all patients maintaining fecal containment for more than half an hour. As for the feeling of incomplete evacuation, 20% reported more than four episodes a day. Antidiarrheal medication was used by 60%, while 76.3% referred food restriction. There was no statistical difference in IPAA function due to disease or to the presence or absence of pelvic complications.

**Figure 2:**
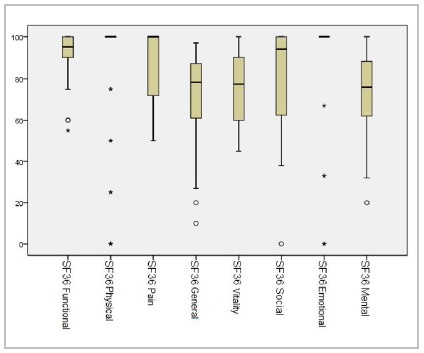
Evaluation of the results of the SF36 questionnaire by domains in patients undergoing PCT with IPAA.

In the assessment of QoL (n = 40), 65% said that the intestinal symptoms had little or no impact on their quality of life, and the average CGQL score was 0.82 and all SF36 domains had median above 70 points (Figure 2).

Neither the disease that led to surgery (FAP or UC) nor the presence of pelvic complications had a statistically significant relationship with these results. Patients with UC also responded the IBDQ questionnaire, which showed good results, with median 180. When evaluating the median by domain, the intestinal one was 57.0 (54.0; 60.0), the systemic IBDQ was 28.0 (24.0; 30.0), the social IBDQ was 30.0 (27.0; 32.0), and the emotional, 65.0 (50.0; 73.0).

Female patients had less bowel movement, less impact of intestinal symptoms on quality of life, and better CGQL scores than men (Table 4).

**Table 4:** Result of the comparison of the ileoanal reservoir function and Quality of Life by sex and education.

	Sex			Education		
	M (n=25)	F (n=15)	P	≤8^th^ (n=17)	>8^th^ (n=22)	p
Bowel						
movements/24 h	7.0	4.0		6.0	5.5	
Median (Q1; Q3)	(5.0; 9.5)	(3.0; 6.0)	0.003^1^	(4.5; 8.5)	(4.0; 7.3)	0.529^1^
Nocturnal bowel						
movements	2.0	1.0		1.0	1.0	
Median (Q1; Q3)	(1.0; 3.0)	(0.0; 1.0)	0.006^1^	(0.5; 2.5)	(0.8; 2.0)	0.616^1^
Escape fecal						
Never	9(36.0)	11 (73.3)	0.155^2^	6 (35.3)	14 (63.6)	0.112^2^
At night	9 (36.0)	2 (13.3)		4 (23.5)	6 (27.3)	
During the day	5 (20.0)	1 (6.7)		5 (29.4)	1 (4.5)	
Both	2 (8.0)	1 (6.7)		2 (11.8)	1 (4.5)	
Incontinence						
Never	21 (84.0)	14 (93.3)	0.633^2^	15 (88.2)	19 (86.4)	1.000^2^
Rarely	4 (16.0)	1 (6.7)		2 (11.8)	3 (13.6)	
Sometimes	0 (0.0)	0 (0.0)		0 (0.0)	0 (0.0)	
Most	0 (0.0)	0 (0.0)		0 (0.0)	0 (0.0)	
Ever	0 (0.0)	0 (0.0)		0 (0.0)	0 (0.0)	
Urgency						
No	15 (60.0)	12 (80.0)	0.298^2^	10 (58.8)	16 (72.7)	0.361^3^
Yes, but retains> half an hour	10 (40.0)	3 (20.0)		7 (41.2)	6 (27.3)	
Yes, between half an hour and five min	0 (0.0)	0 (0.0)	0 (0.0)	0 (0.0)	0 (0.0)	
Yes, less than five minutes	0 (0.0)	0 (0.0)	0 (0.0)	0 (0.0)	0 (0.0)	
Anti-diarrheal						
Yes	10 (40.0)	6 (40.0)	1.000^3^	6 (35.3)	9 (40.9)	0.721^3^
No	15 (60.0)	9 (60.0)		11 (64.7)	13 (59.1)	
Incomplete bowel movements/24 h						
≤1	9 (36.0)	8 (53.3)	0.493^3^	6 (35.3)	11 (50.0)	0.361^2^
>1 e ≤4	11 (44.0)	4 (26.7)		6 (35.3)	9 (40.9)	
>4	5 (20.0)	3 (20.0)		5 (29.4)	2 (9.1)	
Food restriction						
Yes	21 (87.5)	8 (57.1)	0.052^2^	14 (82.4)	14 (70.0)	0.462^2^
No	3 (12.5)	6 (42.9)		3 (17.6)	6 (30.0)	
Worsening QoL						
Nothing or little	12 (48.0)*	14(93.3)**	0.012^1^	10 (58.8)	16 (72.7)	0.262^1^
Sometimes	8 (32.0)	1 (6.7)		3 (17.6)	5 (22.7)	
Much	5 (20.0)	0 (0.0)		4 (23.5)	1 (4.5)	
CGQL	0.77	0.93	0.004^2^	0.80	0.85	0.648^2^
Median (Q1; Q3)	(0.65; 0.90)	(0.83; 0.97)		(0.70; 0.97)	(0.76; 0.93)	
SF36 functional	95.0	100.0		95.0	97.5	
Median (Q1; Q3)	(80.0; 100.0)	(95.0; 100.0)	0.135^2^	(77.5; 100.0)	(93.8; 100.0)	0.069^2^
SF36 physical	100.0	100.0		100.0	100.0	
Median (Q1; Q3)	(87.5; 100.0)	(100.0; 100.0)	0.337^2^	(50.0; 100.0)	(100.0; 100.0)	0.041^2^
SF36 pain	100.0	84.0		72.0	100.0	
Median (Q1; Q3)	(66.5; 100.0)	(72.0; 100.0)	0.988^2^	(61.0; 100.0)	(84.0; 100.0)	0.008^2^
SF36 general	77.0	82.0		62.0	82.0	
Median (Q1; Q3)	(57.0; 87.0)	(62.0; 87.0)	0.492^2^	(52.0; 87.0)	(70.8; 92.0)	0.030^2^
SF36 vitality	70.0	85.0		70.0	80.0	
Median (Q1; Q3)	(55.0; 95.0)	(70.0; 90.0)	0.653^2^	(52.5; 92.5)	(65.0; 91.3)	0.494^2^
SF36 social	88.0	100.0		75.0	100.0	
Median (Q1; Q3)	(50.0; 100.0)	(75.0; 100.0)	0.191^2^	(50.0; 100.0)	(75.0; 100.0)	0.057^2^
SF36 emotional	100.0	100.0		100.0	100.0	
Median (Q1; Q3)	(100.0; 100.0)	(100.0; 100.0)	0.719^2^	(100.0; 100.0)	(91.8; 100.0)	0.183^2^
SF36 mental	68.0	80.0		68.0	80.0	
Median (Q1; Q3)	(48.0; 88.0)	(76.0; 88.0)	0.268^2^	(54.0; 86.0)	(70.0; 88.0)	0.172^2^

^1^Mann Whitney test; ^2^Exact Pearson’s Chi-square test; ^3^Asymptotic Pearson’s Chi-square test. *adjusted residue <= - 1.96 and **adjusted residue> = + 1.96.

Patients with higher education had better SF36 scores in the physical, pain, and general domains, although the assessment of specific IPAA function showed not statistical difference according to the level of education (Table 4).

**Figure 3:**
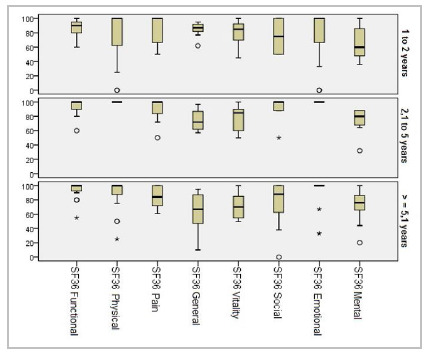
Result of the comparison of the SF36 domains by IPAA time.

Regarding IPAA time, there was no significant difference as to intestinal function, while in QoL evaluation there was worsening in the SF36 general domain with increasing IPAA time (Figure 3).

## DISCUSSION

The IPAA technique revolutionized the surgical treatment of patients with indication for PCT, by avoiding the definitive ileostomy while maintaining the natural evacuation route. It is, however, a surgical procedure with high morbidity, even when performed in reference centers.

In the present study, most patients had a diagnosis of FAP with prophylactic indication for the operation. This is incongruent with most international reference centers, in which 80% to 95% of the population is made up of patients with UC^18^ ^,^ ^22^ ^-^ ^26^.

Being a reference center for more complex surgeries, HC-UFMG constantly receives many cases of FAP. In addition, because of the investigation of family members, the institution has a constant inflow of patients requiring the procedure due to this indication. It can also be speculated that such divergence results from possible resistance in the surgical indication in patients with UC in our environment, with greater insistence on maintaining different modalities of drug treatments in detriment to the surgical one.

In the case of UC, the main surgical indication was clinical intractability, which is in accordance with the literature^27^ ^-^ ^29^, but the use of corticosteroids (94.1%) was higher than in most series, where the rate is 70%^28^. This, associated with the use of biological therapy and the impairment of the patients’ general condition, justified the use of the three-step approach in most patients with UC, which was not observed in patients with FAP.

The diversion of intestinal transit by a protective ileostomy after making the IPAA does not seem to decrease or prevent the formation of IPAA fistulas^30^. On the other hand, it can reduce the clinical consequences of fistulas, which are often disastrous, such as pelvic sepsis^1^. Ileostomy, however, is not a morbidity-free procedure, with dehydration, hydroelectrolytic disorders, enteric fistula in the reconstruction of transit, and intestinal obstruction as some of the complications described^1^. 

Although the occurrence of pelvic sepsis is higher in patients without a protective stoma, complications such as stenosis and IPAA failure tend to occur more in ileostomized patients^31^. However, there is a tendency to indicate the protective stoma for patients at increased risk, such as the elderly, male, in use of higher doses of corticosteroids, and with high body mass index^32^. When these conditions are not present, some centers tend not to perform the protective stoma, carrying out the modified, one or two-stage surgery^33^ ^,^ ^34^.

In the present study, the only case in which no protective ileostomy was performed due to technical difficulties presented a pelvic abscess, with the formation of an IPAA fistula for the vagina. In contrast, the only surgical death occurred due to a fistula in the ileoleal anastomosis after closure of the ileostomy

The surgical mortality of IPAA is about 1%^15^ ^,^ ^18^, and our results (1.8%) are compatible with the literature. The low mortality is justified by the presence of mostly young patients, operations performed in tertiary care center, by a specialized team, and preoperative selection of patients^15^. In our country, Leal et al.^3^ also described low surgical mortality (2.9%) in patients diagnosed with FAP. In this same study, they observed an overall morbidity of 63.8%, the most frequent complications being intestinal obstruction (17.4%), anastomotic stenosis (15.9%), pelvic sepsis (10.1%), IPAA ischemia (4.3%), pouchitis (2.9%), and IPAA fistula to other organs (2.9%).

The morbidity rates published in the literature are quite disparate, ranging from 19% to 62.5%, depending on the criteria used in various studies^15^. The morbidity observed in our study can be considered high, reaching 76.4%. Anastomotic fistula was the most common early complication, its rate of 34.5% being significantly higher than described in the literature. These vary between 5% and 25%^1^ ^,^ ^3^ ^,^ ^15^ ^,^ ^24^ ^,^ ^25^ ^,^ ^30^ ^,^ ^35^. Such heterogeneity is justified in part by the different concepts of fistula, as well as by the experience of the surgical team and the profile of the operated patients. In the present study, we considered the broader concept of fistula, regardless of radiological confirmation, with pelvic abscesses being added to the analysis, to increase diagnosis sensitivity.

Some authors have observed higher rates of septic complications in patients with UC^36^ ^,^ ^37^, which is incongruent with our findings. There was no statistical difference in early complications as to disease type. However, patients with UC had more severe early complications according to the Clavien-Dindo classification, which is justified by the profile of patients with UC, who mostly presented active inflammation and were using immunosuppressive medication, mainly corticosteroids. 

The second most frequent early complication was pelvic hemorrhage, with rates also higher than the literature, which range from 2.4%^38^ to 8%^23^.

Despite the great early surgical morbidity, more than 64.4% of these complications were managed conservatively (Clavien-Dindo I and II), reaching 79.7%, also considering the approaches under local anesthesia, such as puncture guided by image (Clavien-Dindo I, II and IIIa). 

The occurrence of pouchitis and intestinal obstruction were the most common late complications and the rates are in agreement with those reported in the literature^18^ ^,^ ^38^. Pouchitis consists of non-specific inflammation of the IPAA in the absence of intestinal diversion or complications^1^. Although the cause is still unknown, there is recent evidence that IPAA dysbiosis and abnormal mucosal immune response are implicated in the pathogenesis^39^. It presents with symptoms of tenesmus, increased number of bowel movements, fecal leaks, incontinence, urgency, as well as cramps and, occasionally, fever and anal bleeding^39^ ^,^ ^40^. The reports of pouchitis in FAP patients vary between 0 and 10%^3^ ^,^ ^41^, and in the UC these rates reach 40%, in 10 years of IPAA^1^, with an accumulated prevalence of up to 50%^39^, having an important impact on long-term QoL in patients with UC^42^. 

With regard to anastomotic stenosis, we found a lower rate than in other studies, in which they vary from 6.8%^2^ to 20%^43^.

The rate of IPAA fistula to other organs, such as the vagina, is in agreement with the ones of reference centers^15^ ^,^ ^25^, and there is evidence in the literature suggesting a progressive decrease of such complication with the service time experience^25^.

IPAA failure varies in the literature from 2.4%^38^, 6%^15^ to 9%^44^ ^,^ ^45^, which is compatible with our results (9.1%). The main causes of IPAA failure is fistula to the vagina and other pelvic organs and the reservoir septic complications^46^, which are also in line with our findings.

Pelvic complications represent an important part of surgical morbidity, not only due to prevalence and severity, but also due to the potential worsening of IPAA function in the long term. The incidence of pelvic complications ranges from 12%^5^ to 40.8%^47^. Some studies have assessed only the septic pelvic complications and others use a more comprehensive definition, as we did here. Pelvic complications, as well as their multiple approaches, could result in greater fibrosis and pelvic adhesions, with impairment of IPAA accommodativeness and function, and possible QoL deterioration^23^. Though we found a high rate of pelvic complications (47.3%), this did not influence the IPAA function or the QoL. 

The literature is controversial when addressing the impact of pelvic complications on IPAA function and QoL. Recent work has shown an impact on the IBDQ scores in patients with early pelvic complications of Clavien-Dindo grades III and IV^23^. On the other hand, reports state that patients with UC who underwent PCT-IPAA did not have their QoL or IPAA function affected by pelvic complications or by the multiple surgical interventions necessary to treat them^5^ ^,^ ^7^.

The assessment of QoL by IBDQ rendered a total score considered good and compatible with that reported by the literature^48^. In the evaluation by domains, the results were higher than the ones reported by the national literature^9^. Likewise, the values of SF36 by domains in this study were good and compatible with other publications^48^ ^,^ ^49^, with all domains scoring higher than 70.

In a recent publication, a Cleveland Clinic group evaluated the IPAA function and the QoL using clinical parameters and CGQL in 3,707 patients^24^. QoL was excellent for patients with both UC and FAP, regardless of the time of IPAA, data compatible with the results of the present study.

As for the impact of intestinal symptoms on QoL, our results agree with most studies in the literature. Patients have a high degree of satisfaction with the surgery and little impact on quality of life, as reported by most individuals^18^ ^,^ ^20^ ^,^ ^24^ ^,^ ^49^ ^,^ ^50^.

Specifically regarding the IPAA function, the occurrence of six bowel movements in 24 hours with only one at night can be considered quite satisfactory^24^ ^,^ ^25^ ^,^ ^48^ ^,^ ^51^. In addition, most patients can delay bowel movements for more 30 minutes^48^ and deny incontinence for solid stool^24^ ^,^ ^50^. As for the presence of fecal leak and incomplete evacuation, our data are also quite like those of other authors. Fecal leak is present in 40% to 50% of patients^24^ ^,^ ^25^ ^,^ ^48^, while in 25%^25^ this escape is nocturnal, and approximately 60% of patients exhibit more than one episode of incomplete evacuation a day^48^.

Regarding the assessment of QoL and IPAA function, there was no difference between patients with UC or FAP, although we would expect a greater impact on QoL of patients with FAP, since such patients do not live with chronic symptoms related to the presence of intestinal inflammation, as usually occurs in those with UC. Other authors also did not observe any functional or QoL difference when analyzing patients with FAP or UC undergoing IPAA^18^ ^,^ ^52^.

Female patients admittedly have worse IPAA function, displaying more urgency and bowel movements in 24 hours^48^, increased nighttime frequency, and daytime incontinence^53^, in addition to worse QoL among those who became pregnant and delivered after the IPAA^42^. It is believed that there is an influence of other factors that justify these results, such as the number and route of delivery or the occurrence of obstetric injuries, although this has not yet been adequately evaluated in high-impact studies^48^ ^,^ ^54^. However, the present study found the opposite, with women presenting better CGQL scores, reports of lesser interference of intestinal symptoms with QoL, fewer nighttime and 24-hour bowel movements, and a tendency to report less food restriction. The selection of patients for IPAA may have strongly influenced these results, as in our sample most women were young, nulliparous or with up to two vaginal deliveries. Other factors, such as greater care with own health^55^ ^-^ ^57^ and dietary habits^58^ ^,^ ^59^ contribute to better functional results in females.

Although education does not influence IPAA function, patients with a higher educational level showed better results in the evaluation by SF36 in the general, physical, and pain domains, in addition to a tendency for better results also in the functional and social domains. Higher education implies not only better knowledge of the disease itself and the surgical procedure the patient has undergone, but also results in a greater financial return on work activities, an increased sense of personal control, and freedom of decision^52^ ^,^ ^60^ ^,^ ^61^. These factors contribute to a greater capacity for adaptation^52^ ^,^ ^61^ after IPAA, which would result in fewer functional limitations.

We observed no functional changes related to the time elapsed after the IPAA, probably due to the small sample size. Such results are quite variable, mainly due to the different periods evaluated. Cleveland Clinic authors^18^ ^,^ ^24^ demonstrated stable IPAA function even after ten years of follow-up. On the other hand, the opposite has been noted in studies with 20 or more years of follow up48, suggesting function deterioration, with increased nighttime frequency, fecal leaks, need for antidiarrheic medications, and use of hygienic protection^51^. In all of these studies^24^ ^,^ ^48^ ^,^ ^51^, despite function worsening, the QoL as measured by the CGQL and SF36 scores remained unchanged and at high levels over time.

Our results also show high, stable QoL, with worsening only in the overall SF36, with statistically significant difference between groups with one and two years of IPAA compared with those with more than five years. This result was not consistent with the literature and may have been influenced by the sample size or age and comorbidities in the group with longer IPAA time.

This study presents limitations, the absence of sample calculation and sample size being the main ones, not allowing for more stratification of patients, and possibly interfering with the results statistical analysis. In addition, the evaluation of surgical results was performed by review of medical records, which may generate some bias in data collection and interpretation.

Another limiting factor was the absence of cross cultural validation of the main IPAA function evaluation questionnaires in our midst. The three IPAA function questionnaires most used in the literature are the Function Oresland Score^21^, the Pouch Function Score^19^, and the Pouch Disfunction Score^20^. All questionnaires score the different complaints related to the IPAA and generate a result on a numerical scale. The higher the result, the worse the IPAA’s function at all scales. In the absence of their cross cultural validation, there was an evaluation of all items separately, without generating numerical results, which would certainly facilitate the evaluation of the obtained data and the comparison with those published in the literature. 

On the other hand, it was possible to present a detailed survey of surgical, functional, and QoL results in patients undergoing IPAA, from a national sample, considering that this type of procedure is still relatively little performed in our country. Thus, we hope to contribute to the increase in the indication of this type of procedure, as well as to a better understanding of complications and correlation with the functional and QoL-related results. 

We expect that other national studies, preferably multi-institutional, may bring more robust results in terms of functional and QoL results, in addition to being able to validate the specific questionnaires of IPAA function, thus facilitating the postoperative monitoring of patients and the comparison with the international literature data.

## CONCLUSION

PCT-IPAA has low mortality and high morbidity, the most serious early complications occurring more frequently in patients with UC. Despite this, the IPAA function and QoL can be considered satisfactory, regardless of the type of disease and the occurrence of pelvic complications.

Female patients display better IPAA function and QoL. However, patients with higher education levels, despite having the same IPAA function, have better QoL scores in the physical, pain, and general domains of the SF36. Patients with longer IPAA time present worse results only in the general domain of SF36.

## References

[1] Sagar PM, Pemberton JH (2012). Intraoperative, postoperative and reoperative problems with ileoanal pouches. Br J Surg.

[2] de Zeeuw S, Ahmed Ali U, Donders RA, Hueting WE, Keus F, van Laarhoven CJ, Update of complications and functional outcome of the ileo-pouch anal anastomosis: overview of evidence and meta-analysis of 96 observational studies (2012). Int J Colorectal. Dis.

[3] Leal RF, Ayrisono MLS, Coy CSR Fagundes JJ, Góes JRN (2008). Complicações imediatas e tardias após cirurgia de reservatório ileal na polipose adenomatosa familiar. Arq. gastroenterol.

[4] Ourô S, Thava B, Shaikh I, Clark SK (2016). Management of pouch dysfunction in a tertiary centre. Colorectal Dis.

[5] Hallberg H, Ståhlberg D, Akerlund JE (2005). Ileal pouch-anal anastomosis (IPAA) functional outcome after postoperative pelvic sepsis. A prospective study of 100 patients. Int J Colorectal Dis.

[6] Chessin DB, Gorfine SR, Bub DS, Royston A, Wong D, Bauer JJ (2008). Septic complications after restorative proctocolectomy do not impair functional outcome long-term follow-up from a specialty center. Dis Colon Rectum.

[7] Mennigen R, Senninger N, Bruewer M, Rijcken E (2012). Pouch function and quality of life after successful management of pouch-related septic complications in patients with ulcerative colitis. Langenbecks Arch Surg.

[8] Selvaggi F, Sciaudone G, Limongelli P, Di Stazio C, Guadagni I, Pellino G (2010). The effect of pelvic septic complications on function and quality of life after ileal pouch-anal anastomosis a single center experience. Am Surg.

[9] Tilio MSG, Arias LB, Camargo MG, Oliveria PSP, Ayrizono MLS (2013). Quality of life in patients with ileal pouch for ulcerative colitis. J Coloproctol (Rio J).

[10] Teixeira MG, Ponte ACA, Sousa M, Almeida MG, Silva E, Calache JE (2003). Short- and long-term outcomes of ileal pouch-anal anastomosis for ulcerative colitis. Rev Hosp Clin.

[11] Meyer AL, Teixeira MG, Almeida MG, Kiss DR, Nahas SC, Cecconello I (2009). Quality of life in the late follow-up of ulcerative colitis patients submitted to restorative proctocolectomy with sphincter preservation over ten years ago. Clinics.

[12] Campos FG, Real Martinez CA, Monteiro de Camargo MG, Cesconetto DM, Nahas SC, Cecconello I (2018). Laparoscopic Versus Open Restorative Proctocolectomy for Familial Adenomatous Polyposis. J Laparoendosc Adv Surg Tech A.

[13] Moreira LF, Pessoa MCM, Mattana DS, Schmitz FF, Volkweis BS, ANtoniazzi JL (2016). Adaptação cultural e teste da escala de complicações cirúrgicas de Clavien-Dindo traduzida para o Português do Brasil. Rev Col Bras Cir.

[14] Rahbari NN, Weitz J, Hohenberger W, Heald RJ, Moran B, Ulrich A (2010). Definition and grading of anastomotic leakage following anterior resection of the rectum a proposal by the International Study Group of Rectal Cancer. Surgery.

[15] Gorgun E, Remzi FH (2004). Complications of ileoanal pouches. Clin Colon Rectal Surg.

[16] Ciconelli RM, Ferraz MB, Santos W, Meinão I, Quaresma MR (1999). Tradução para a língua portuguesa e validação do questionário genérico de avaliação de qualidade de vida SF-36 (Brasil SF-36). Rev Bras Reumatol.

[17] Pontes RMA, Miszputen SJ, Ferreira OF, Miranda C, Ferraz MB (2004). Qualidade de vida em pacientes portadores de doença inflamatória intestinal tradução para o português e validação do questionário "Inflammatory Bowel Disease Questionnaire" (IBDQ). Arqu Gastroenterol.

[18] Fazio VW, Ziv Y, Church JM, Oakley JR, Lavery IC, Milsom JW (1995). Ileal pouch-anal anastomoses complications and function in 1005 patients. Ann Surg.

[19] Lovegrove RE, Fazio VW, Remzi FH, Tilney HS, Nicholls RJ, Tekkis PP (2010). Development of a pouch functional score following restorative proctocolectomy. Br J Surg.

[20] Brandsborg S, Nicholls RJ, Mortensen LS, Laurberg S (2013). Restorative proctocolectomy for ulcerative colitis development and validation of a new scoring system for pouch dysfunction and quality of life. Colorectal Dis.

[21] Oresland T, Fasth S, Nordgren S, Hultén L (1989). The clinical and functional outcome after restorative proctocolectomy A prospective study in 100 patients. Int J Colorectal Dis.

[22] Baek SJ, Dozois EJ, Mathis KL, Lightner AL, Boostrom SY, Cima RR (2016). Safety, feasibility, and short-term outcomes in 588 patients undergoing minimally invasive ileal pouch-anal anastomosis a single-institution experience. Tech Coloproctol.

[23] McCombie A, Lee Y, Vanamala R, Gearry R, Frizelle F, McKay E (2016). Early postoperative complications have long-term impact on quality of life after restorative proctocolectomy. Medicine (Baltimore).

[24] Fazio VW, Kiran RP, Remzi FH, Coffey JC, Heneghan HM, Kirat HT (2013). Ileal pouch anal anastomosis analysis of outcome and quality of life in 3707 patients. Ann Surg.

[25] Remzi FH, Lavryk OA, Ashburn JH, Hull TL, Lavery IC, Dietz DW (2017). Restorative proctocolectomy an example of how surgery evolves in response to paradigm shifts in care. Colorectal Dis.

[26] Kjaer MD, Laursen SB, Qvist N, Kjeldsen J, Poornoroozy PH (2014). Sexual function and body image are similar after laparoscopy-assisted and open ileal pouch-anal anastomosis. World J Surg.

[27] Rencuzogullari A, Stocchi L, Costedio M, Gorgun E, Kessler H, Remzi FH (2017). Characteristics of learning curve in minimally invasive ileal pouch-anal anastomosis in a single institution. Surg Endosc.

[28] Uchino M, Ikeuchi H, Sugita A, Futami K, Watanabe T, Fukushima K, Tatsumi K, Koganei K, Kimura H, Hata K, Takahashi K, Watanabe K, Mizushima T, Funayama Y, Higashi D, Araki T, Kusunoki M, Ueda T, Koyama F, Itabashi M, Nezu R, Suzuki Y, a research grant on intractable disease affiliated with the Japan Ministry of Health Labor Welfare (2018). Pouch functional outcomes after restorative proctocolectomy with ileal-pouch reconstruction in patients with ulcerative colitis Japanese multi-center nationwide cohort study. J Gastroenterol.

[29] Germain A, de Buck van Overstraeten A.Wolthuis A.Ferrante M.Vermeire S.Van Assche G (2018). Outcome of restorative proctocolectomy with ileo-anal pouch for ulcerative colitis effect of changes in clinical practice. Colorectal Dis.

[30] Sahami S, Buskens CJ, Fadok TY, Tanis PJ, de Buck van Overstraeten A, Wolthuis AM (2016). Defunctioning Ileostomy is not Associated with Reduced Leakage in Proctocolectomy and Ileal Pouch Anastomosis Surgeries for IBD. J Crohns Colitis.

[31] Weston-Petrides GK, Lovegrove RE, Tilney HS, Heriot AG, Nicholls RJ, Mortensen NJ (2008). Comparison of outcomes after restorative proctocolectomy with or without defunctioning ileostomy. Arch Surg.

[32] Remzi FH, Fazio VW, Gorgun E, Ooi BS, Hammel J, Preen M (2006). The outcome after restorative proctocolectomy with or without defunctioning ileostomy. Dis Colon Rectum.

[33] Kirat HT, Remzi FH (2010). Technical aspects of ileoanal pouch surgery in patients with ulcerative colitis. Clin Colon Rectal Surg.

[34] Zittan E, Wong-Chong N, Ma GW, McLeod RS, Silverberg MS, Cohen Z (2016). Modified Two-stage Ileal Pouch-Anal Anastomosis Results in Lower Rate of Anastomotic Leak Compared with Traditional Two-stage Surgery for Ulcerative Colitis. J Crohns Colitis.

[35] Ministério da Educação (BR). Conselho Nacional da Educação (2010). Resolução CNE/CEB 7/2010. Fixa Diretrizes Curriculares Nacionais para o Ensino Fundamental de 9 (nove) anos.

[36] Heuschen UA, Hinz U, Allemeyer EH, Autschbach F, Stern J, Lucas M (2002). Risk factors for ileoanal j pouch-related septic complications in ulcerative colitis and familial adenomatous polyposis. Ann Surg.

[37] Cotte E, Mohamed F, Nancey S, François Y, Glehen O, Flourié B (2011). Laparoscopic total colectomy Does the indication influence the outcome?. World J Gastrointest Surg.

[38] Dafnis G (2016). Early and late surgical outcomes of ileal pouch-anal anastomosis within a defined population in Sweden. Eur J Gastroenterol Hepatol.

[39] Angriman I, Scarpa M, Castagliuolo I (2014). Relationship between pouch microbiota and pouchitis following restorative proctocolectomy for ulcerative colitis. World J Gastroenterol.

[40] Hata K, Ishihara S, Nozawa H, Kawai K, Kiyomatsu T, Tanaka T (2017). Pouchitis after ileal pouch-anal anastomosis in ulcerative colitis Diagnosis, management, risk factors, and incidence. Dig Endosc.

[41] Lovegrove RE, Tilney HS, Heriot AG, von Roon AC, Athanasiou T, Church J (2006). A comparison of adverse events and functional outcomes after restorative proctocolectomy for familial adenomatous polyposis and ulcerative colitis. Dis Colon Rectum.

[42] Coffey JC, Winter DC, Neary P, Murphy A, Redmond HP, Kirwan W (2002). Quality of life after ileal pouch-anal anastomosis an evaluation of diet and other factors using the Cleveland Global Quality of Life instrument. Dis Colon Rectum.

[43] Ganschow P, Warth R, Hinz U, Büchler MW, Kadmon M (2014). Early postoperative complications after stapled vs handsewn restorative proctocolectomy with ileal pouch-anal anastomosis in 148 patients with familial adenomatous polyposis coli a matched-pair analysis. Colorectal Dis.

[44] Meagher AP, Farouk R, Dozois RR, Kelly KA, Pemberton JH (1998). J ileal pouch-anal anastomosis for chronic ulcerative colitis complications and long-term outcome in 1310 patients. Br J Surg.

[45] Mark-Christensen A, Brandsborg S, Laurberg S (2018). Primary fecal diversion and bowel dysfunction in restorative proctocolectomy for ulcerative colitis a nationwide cross-sectional study. Int J Colorectal Dis.

[46] Fazio VW, Tekkis PP, Remzi F, Lavery IC, Manilich E, Connor J (2003). Quantification of risk for pouch failure after ileal pouch anal anastomosis surgery. Ann Surg.

[47] Noh GT, Han J, Cho MS, Hur H, Min BS, Lee KY (2017). Factors affecting pouch-related outcomes after restorative proctocolectomy. PLoS One;.

[48] Brandsborg S, Tøttrup A, Nicholls J, Laurberg S (2013). Restorative proctocolectomy in patients with ulcerative colitis a cross-sectional Danish population study on function and quality of life. Colorectal Dis.

[49] Fazio VW, O'Riordain MG, Lavery IC, Church JM, Lau P, Strong SA (1999). Long-term functional outcome and quality of life after stapled restorative proctocolectomy. Ann Surg.

[50] de Buck van Overstraeten A, Wolthuis AM, Vermeire S, Van Assche G, Laenen A, Ferrante M (2014). Long-term functional outcome after ileal pouch anal anastomosis in 191 patients with ulcerative colitis. J Crohns Colitis.

[51] Lorenzo G, Maurizio C, Maria LP, Tanzanu M, Silvio L, Mariangela P (2016). Ileal pouch-anal anastomosis 20 years later is it still a good surgical option for patients with ulcerative colitis?. Int J Colorectal Dis.

[52] Ross CE, Van Willigen M (1997). Education and the Subjective Quality of Life. J Health Soc Behav.

[53] Lightner AL, Mathis KL, Dozois EJ, Hahnsloser D, Loftus EV, Raffals LE (2017). Results at Up to 30 Years After Ileal Pouch-Anal Anastomosis for Chronic Ulcerative Colitis. Inflamm Bowel Dis.

[54] Chang S, Shen B, Remzi F (2017). When Not to Pouch Important Considerations for Patient Selection for Ileal Pouch-Anal Anastomosis. Gastroenterol Hepatol (N Y).

[55] Costa-Júnior FM, Maia ACB (2009). Concepções de homens hospitalizados sobre a relação entre gênero e saúde. Psic.: Teor. e Pesq.

[56] Gomes R, Moreira MCN, Nascimento EF, Rebello LEFS, Couto MT, Schraiber LB (2011). Os homens não vêm Ausência e/ou invisibilidade masculina na atenção primária. Ciênc Saúde Coletiva.

[57] Thompson AE, Anisimowicz Y, Miedema B, Hogg W, Wodchis WP, Aubrey-Bassler K (2016). The influence of gender and other patient characteristics on health care-seeking behaviour a QUALICOPC study. BMC Fam Pract.

[58] Malta DC, Andrade SSCA, Stopa SR, Pereira CA, Szwarcwald CL, Silva JB, Estilos de vida da população brasileira: resultados da Pesquisa Nacional de Saúde.2013 (2015). Epidemiolo Serv. Saúde.

[59] Assumpção D, Domene SMA, Fisberg RM, Canesqui AM, Barros MBA (2017). Diferenças entre homens e mulheres na qualidade da dieta estudo de base populacional em Campinas, São Paulo. Ciênc Saúde Coletiva.

[60] Israel BA, Checkoway B, Schulz A, Zimmerman M (1994). Health education and community empowerment conceptualizing and measuring perceptions of individual, organizational, and community control. Health Educ Q.

[61] Zajacova A, Lawrence EM (2018). The relationship between education and health reducing disparities through a contextual approach. Annu Rev Public Health.

